# Identification of Immune Subtypes of Esophageal Adenocarcinoma to Predict Prognosis and Immunotherapy Response

**DOI:** 10.3390/ph15050605

**Published:** 2022-05-14

**Authors:** Chen Ling, Xiuman Zhou, Yanfeng Gao, Xinghua Sui

**Affiliations:** School of Pharmaceutical Sciences (Shenzhen), Sun Yat-sen University, Shenzhen 518107, China; lingch6@mail2.sysu.edu.cn (C.L.); zhouxm36@mail.sysu.edu.cn (X.Z.)

**Keywords:** esophageal adenocarcinoma, immune checkpoint inhibitors, molecular subtypes, immunotherapy response

## Abstract

A low response rate limits the application of immune checkpoint inhibitors (ICIs) in the treatment of esophageal adenocarcinoma (EAC), which requires the precise characterization of heterogeneous tumor microenvironments. This study aimed to identify the molecular features and tumor microenvironment compositions of EAC to facilitate patient stratification and provide novel strategies to improve clinical outcomes. Here, we performed consensus molecular subtyping with nonnegative matrix factorization (NMF) using EAC data from the Cancer Genome Atlas (TCGA) and identified two distinct subtypes with significant prognostic differences and differences in tumor microenvironments. The findings were further validated in independent EAC cohorts and potential response to ICI therapy was estimated using Tumor Immune Dysfunction and Exclusion (TIDE) and SubMap methods. Our findings suggest that EAC patients of subtype 2 with low levels of cancer-associated fibroblasts, tumor associated macrophages (TAMs), and MDSCs in the tumor microenvironment may benefit from PD-1 blockade therapy, while patients of subtype 1 are more responsive to chemotherapy or combination therapy. These findings might improve our understanding of immunotherapy efficacy and be useful in the development of new strategies to better guide immunotherapy and targeted therapy in the treatment of EAC.

## 1. Introduction

Esophageal cancer is the sixth most prevalent cause of cancer-related death, with a 5-year survival rate of 14% [1]. Esophageal adenocarcinoma (EAC) and squamous cell carcinoma (ESCC) are the two main histological types of esophageal cancer. EAC usually occurs in the lower middle of the esophagus [2], and its incidence is increasing in Western countries [3]. Current available treatments for EAC include surgery, perioperative chemotherapy [4,5], neoadjuvant chemoradiotherapy [6], and targeted therapies against growth factors EGFR, VEGFR, HER-2 [7] and DNA methyltransferases [8]. However, esophageal cancer lacks common oncogenic driver mutations [9] and conventional treatments have a limited efficacy with relatively severe adverse effects. So far, only the targeting of HER-2 positive tumors has shown clinical efficacy to a certain extent [8,10], while other therapies targeting oncogenes or related pathways have proven mostly ineffective due to drug resistance [11]. Recent advancements in nanoparticle-based drug delivery systems have provided novel therapeutic approaches for improving cellular targeting, reducing side effects, and combating drug resistance [12,13,14]. Although significant progress has been made in molecularly profiling esophageal cancer to identify patient subgroups who might benefit from targeted therapy [15], overall therapeutic success remains limited. Thus, molecular subtyping of EAC is urgently needed to facilitate patient stratification and pharmaceutical development.

Recently, immunotherapy—especially immune checkpoint inhibitors (ICIs) targeting programmed death receptor 1 (PD-1) and its ligand (PD-L1)—has provided a promising new avenue for treating esophageal cancer [16,17]. With the rise of immunotherapy, the treatment of esophageal cancer has also moved towards the precision medicine era. However, employing PD-1/PD-L1 antibodies alone or in combination with other ICIs to target the immunological microenvironment has yielded inconsistent results. For example, the overall response rate reached 40% in PD-L1-positive EAC patients in the KEYNOTE-028 trial [18]. In contrast, only 5.2% of patients with advanced or metastatic EAC after two standard therapies achieved partial response, and no patients achieved complete response in the phase II KEYNOTE-180 trial [19]. Pembrolizumab showed no overall survival (OS) advantage over chemotherapy for EAC patients [20]. An elucidation of the potential mechanism regulating ICI response and a classification of the most sensitive subpopulations of EAC patients are still lacking. Reliable predictive biomarkers are thus required to identify potential responders to ICIs therapy [21].

It is vital to further develop reliable and predictive biomarkers for prospective ICI response in order to increase the practicability of ICI therapy. There have been many studies on predictive biomarkers, including tumor mutation burden (TMB), microsatellite instability (MSI), and PD-L1 status [22,23]. A previous study classified gastrointestinal adenocarcinomas of the upper (gastroesophagus) and lower digestive tract (colorectal) into subgroups of chromosomal instability, microsatellite instability, and hypermethylation phenotype [24]. A pan-cancer analysis of TCGA also identified six immunogenomic subtypes across 33 cancer types [25]. However, MSI was rarely observed in EAC [26] and the association of TMB with overall survival was not observed after excluding MSI-high patients [27]. Further molecular and immune subtyping is needed to guide clinical treatment for EAC patients. There is mounting evidence that the tumor microenvironment (TME) is closely related to prognosis and therapy response. Studies have also suggested that immunotherapy response is highly dependent on dynamic tumor–immune interactions in the TME [28,29]. A deeper understanding of the TME could provide new opportunities to identify novel therapeutic targets for immunotherapy [30,31].

Since the TME plays a critical role in determining immunotherapy response, we hypothesize that EAC can be further separated into subtypes with distinct immunological states which may provide guidance for individualized patient treatment. In this study, we performed integrated bioinformatic analyses to identify EAC subtypes with distinguished prognoses and therapeutic responses. Specifically, we comprehensively characterized the gene expression patterns, functional annotations, and immune landscapes of the identified EAC subtypes. We then predicted their sensitivity in responding to chemotherapeutic drugs and ICIs. Finally, we constructed and validated a subtype classifier based on molecular and immunological features of EAC subtypes.

## 2. Results

### 2.1. Identification of EAC Subtypes

**Figure 1:**
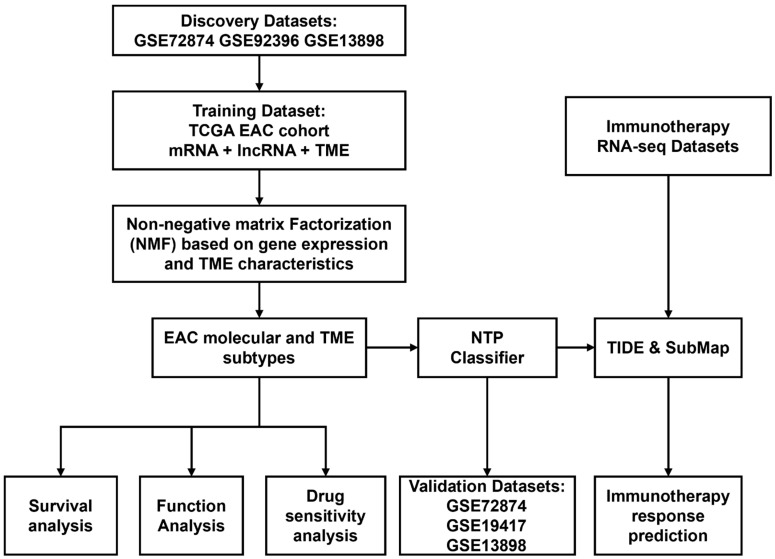
Flow chart of this study.

The study’s workflow is shown in Figure 1. Considering the limited size of the TCGA-EAC cohort, we combined a cohort of 189 samples from GSE72874, GSE92396, and GSE13898, including 54 peritumor esophageal samples and 135 esophageal adenocarcinoma samples after removing potential batch effects. In total, 276 protein-coding genes were identified as differentially expressed. They were further combined with DEGs in the TCGA-EAC cohort as candidate genes for identifying subtypes in the following clustering.

We applied eight algorithms to characterize the TME composition of EAC using the R package IOBR. Among them, CIBERSORT is the most widely used method for enumerating 22 immune cell types in TME. TIMER quantifies six tumor-infiltrating immune cell types. ESTEMATE dissects both stromal and immune signatures to determine tumor purity. The quanTIseq method enumerates 10 immune cell types from bulk RNA-seq data. IPS estimates 28 tumor-infiltrating lymphatic cell types, including effector and memory T cells and immunosuppressive cells. MCP-counter estimated eight immune and two stromal cell types. By default, xCell provides a comprehensive view of 64 immune and stromal cell types. By comparing the gene expression profiles of specific cells, EPIC decodes the proportions of immune and cancer cells. In all, 137 TME-related scores were used as candidate features for the following subtype clustering.

**Figure 2:**
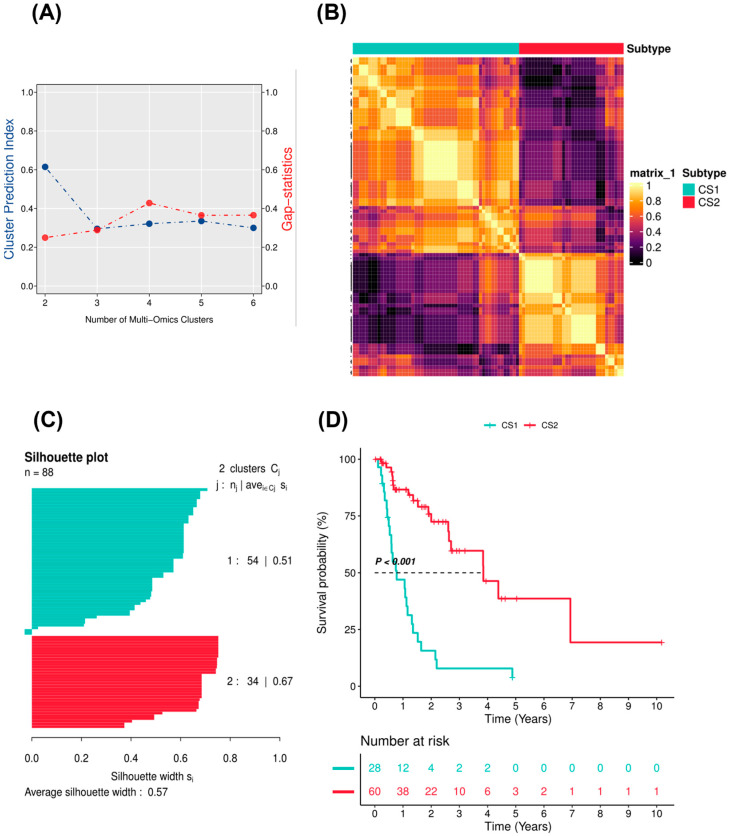
Identification of EAC subtypes using IntNMF and consensus clustering. (**A**) Identification of optimal cluster numbers by calculating CPIs (blue line) and gap statistics (red line) in TCGA-EAC. (**B**) Consensus heatmap based on results from 10 multi-omics integrative clustering algorithms with a cluster number of 2. (**C**) Quantification of sample similarity using silhouette scores based on consensus ensemble results. (**D**) Kaplan–Meier survival curve of EAC subtypes in TCGA-EAC.

Further filtering procedures were performed on the TCGA EAC cohort before IntNMF clustering using the R package MOVICS. Genes with a significant prognostic value (*p* < 0.05 according to univariate cox regression) and TME scores with median absolute deviation (MAD) values greater than 0.5 were retained. MAD statistics provided a robust and effective tool with which to filter features for clustering and differential gene expression analysis, even with little information [32]. In the end, the expression profiles of 1537 protein-coding mRNA genes, 463 long non-coding RNA genes, and 30 TME scores were used to identify EAC subtypes. According to the clustering prediction index (Figure 2A), the optimal number of clusters (k) was 2. Gap statistics did not decline too much at a k of 2. Considering the relatively limited cohort size of the training cohort, a k of 2 was chosen as the optimal clustering number for further analyses. In addition, we calculated the consensus matrix by integration of the clustering results derived from another nine algorithms (including SNF, PINSPlus, NEMO, COCA, LRAcluster, ConsensusClustering, iClusterBayes, CIMLR, and MoCluster) to improve clustering robustness. The consensus matrix represented the probability of each sample belonging to the same subtype using different clustering methods. The diagonal rectangle illustrated in the consensus heatmap (Figure 2B) suggested the stability of the two subtypes. Silhouette values were also calculated for each sample to determine the extent of similarity within clusters compared to other clusters. The average silhouette width was 0.57 across all the 10 clustering algorithms, suggesting the robustness of the EAC subtypes (Figure 2C). Survival analysis showed that the prognostic difference between the two subtypes was significant (*p* < 0.001; Figure 2D). Stratified survival analyses based on the age and clinical stage subgroups indicated the prognostic value of the two EAC subtypes (Supplementary Figure S1).

### 2.2. Molecular and TME Characteristics of EAC Subtypes

**Figure 3:**
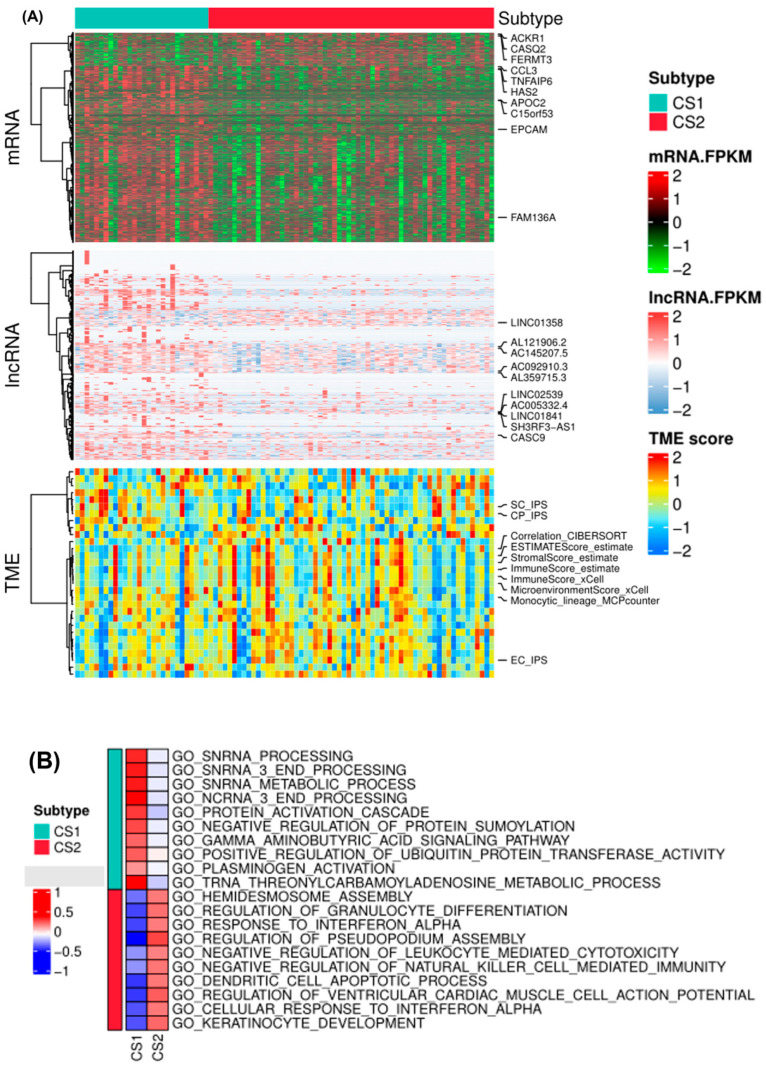

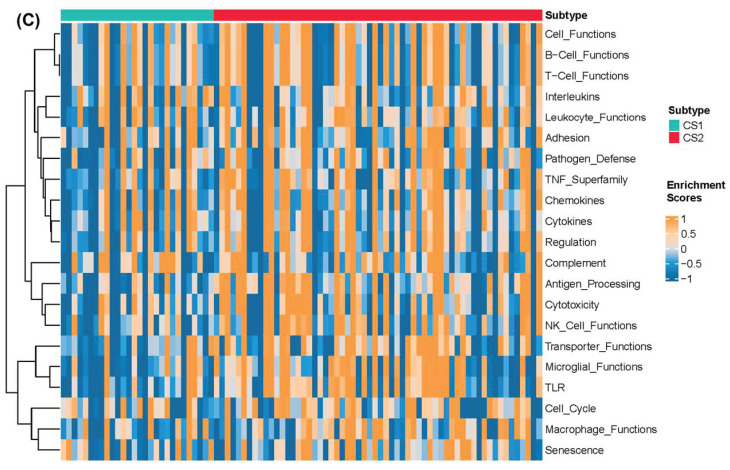
Molecular and TME characteristics and enrichment pathways of EAC subtypes with clinicopathological annotations. (**A**) Heat map for mRNAs, lncRNAs, and TME scores for the EAC subtypes with clinical characteristics. (**B**) GO enrichment analyses of upregulated genes differentially expressed in the EAC subtypes of the TCGA-EAC cohort. SC_IPS: suppressor cell score in IPS (immunophenoscores); CP_IPS: checkpoint or immunomodulator score in IPS; EC_IPS: effector cell score in IPS. (**C**) Heatmap of enrichment scores of the gene set of interest for EAC subtypes in the TCGA-EAC cohort.

Subtype-specific mRNA, lncRNA, and TME scores were identified with a false discovery rate (FDR) of less than 0.01 and a log2 fold change greater than 1. A total of 938 and 97 features were identified in subtype 1 and subtype 2, respectively. Upregulated differentially expressed protein-coding mRNAs in subtype 2 included ACKR1, CASQ2, FERMT3, EPCAM, among others. DEGs such as CCL3, TNFAIP6, HAS2, and APOC2 were upregulated in EAC subtype 1. Differentially expressed lncRNAs, such as LINC01358, AL121906.2, AC092910.3, AC005332.4, and CASC9, were mostly upregulated in EAC subtype 1. TME scores showed mixed characteristics for the two subtypes. Both subtypes contained high levels of suppressor cells and checkpoint expression (SC_IPS and CP_IPS by immunophenoscores), and some samples showed high stromal scores and immune scores by ESTIMATE and xCell algorithms (Figure 3A).

**Figure 4:**
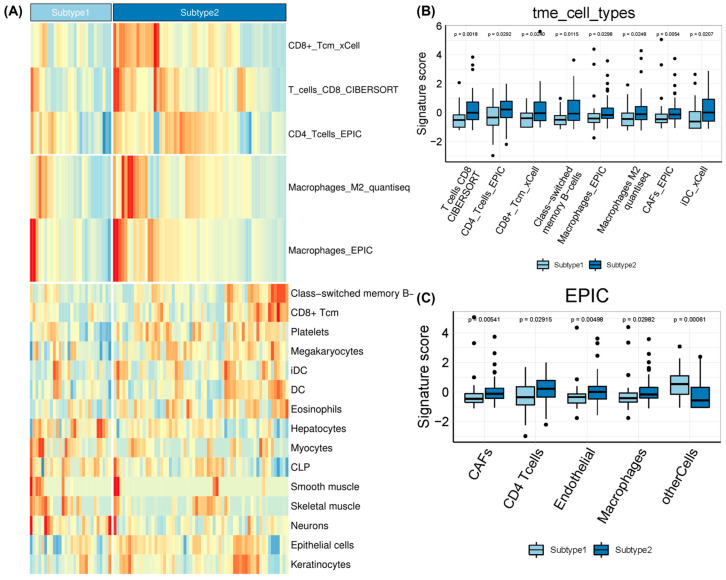
The tumor microenvironment landscape of EAC subtypes. (**A**) Heatmap of significantly different TME cell types between the two EAC subtypes. (**B**) Boxplot of TME cell type signature scores obtained using CIBERSORT, EPIC, xCell, and quanTIseq methods. (**C**) TME signature scores estimated by EPIC.

Subsequently, a GO enrichment analysis of biological processes was conducted based on DEGs for the subtypes (Figure 3B). EAC subtype 2 was significantly enriched in the ‘negative regulation of leukocyte mediated cytotoxicity’, ‘negative regulation of natural killing (NK) cell mediated immunity’, ‘hemidesmosome activity’, ‘gamma aminobutyric acid signaling pathway’, and ‘keratinocyte development’ pathways. Gene signatures of EAC subtypes were mainly enriched in immune, metabolic and T cell-related processes, but the enrichment scores showed the opposite according to the relative expression levels of the signature genes. We further calculated the enrichment score of each sample based on a list of genes of interest using gene set variation analysis (Figure 3C). By combining CIBERSORT, EPIC, and quanTIseq algorithms, we identified significantly different TME signatures between the two EAC subtypes and found that EAC subtype 2 had higher levels of CD4^+^ and CD8^+^ T cell infiltration as well as M2 macrophages and cancer-associated fibroblasts (Figure 4). Kaplan–Meier survival analyses also revealed prognostic significance for mast cells, eosinophils, megakaryocytes, Th2 cells, and B cells in the TCGA-EAC cohort (Supplementary Figure S2). EAC subtype 2 had significantly increased immune cell infiltration (T cell, B cell, and NK cell function) and antigen-processing activities, while subtype 1 had significantly decreased macrophage function and transporter functions. Collectively, the two EAC subtypes displayed distinct gene expression patterns and immune microenvironments.

### 2.3. Therapeutic Sensitivity of EAC Subtypes

**Figure 5:**
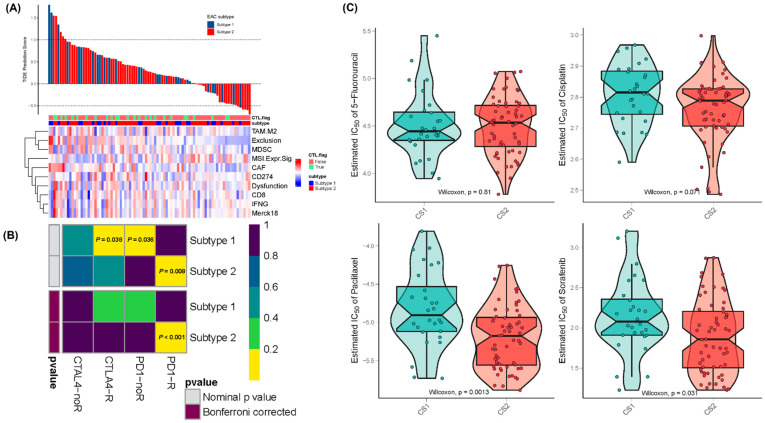
Differential immunotherapeutic and chemotherapeutic responses of EAC subtypes. (**A**) A waterfall plot of TIDE prediction scores across 88 TCGA-EAC tumor samples and a heatmap of TIDE reported signature scores. Dysfunction: T cell dysfunction potential of the tumor; Exclusion: T cell exclusion potential of the tumor; CAF: Pearson correlation between input expression profiles and FAP^+^ cancer associated fibroblast (CAF) signatures; TAM.M2: Pearson correlation between input expression profiles and TAM (M2 subtype) signatures; CTL.flag: Tumor category defined by the level of CTL (Cytotoxic T Lymphocyte)—high: True or low: False; Merck18: T cell-inflamed siganture (PMID: 28650338); MSI Expr Sig: MSI prediction model trained from TCGA STAD Cancer through Ridge Regression; MDSC: Pearson correlation between input expression profiles and MDSC signatures. (**B**) SubMap analysis manifested that the EAC subtype 2 could be more sensitive to the PD1 inhibitor (Bonferroni-corrected *p* < 0.001). (**C**) The boxplots of the estimated IC_50_ values for 5-Fluorouracil, Cisplatin, Paclitaxel, and Sorafenib are shown.

Recently, studies have found that the ICIs nivolumab (CheckMate 577 and CheckMate 649) and pembrolizumab in combination with chemotherapy (KEYNOTE-590) have achieved revolutionary response rates in esophageal cancer [33]. We therefore applied the TIDE algorithm to predict the probability of response to ICI immunotherapy in EAC patients. TIDE is a computational framework developed to assess the potential of tumor immune escape from gene expression profiles by modeling T cell dysfunction and exclusion in tumors (Figure 5A). Prediction of non-response to ICI immunotherapy was based on a combination of TIDE and IFNG signatures. The results demonstrated that subtype 2 (30%, 18/60) contained more responders compared to subtype 1 (21.4%, 6/28), but the difference was not significant (Fisher’s exact test, *p* = 0.4). The TIDE prediction score can be interpreted as a z-score; a higher value indicates a higher potential of tumor immune evasion and a lower likelihood of benefitting from anti-PD-1/CTLA-4. High TIDE scores and worse ICI responses were positively correlated with cancer-associated fibroblast (CAF) signatures, M2 subtype TAM signatures, MDSC signatures, and T cell-inflamed signatures (Merck18) [34].

We further used the SubMap algorithm to compare the expression profiles of EAC subtypes with a published dataset containing 47 melanoma patients who responded to immunotherapies [35]. The results indicated that EAC subtype 2 was more likely to respond to anti-PD-1 therapy (Bonferroni-corrected *p* < 0.001) (Figure 5B).

Considering that chemotherapy and neoadjuvant chemoradiotherapy are the dominant treatment strategies for esophageal cancer, we also assessed the response of EAC subtypes to four commonly used chemo drugs—5-fluorouracil, cisplatin, paclitaxel, and sorafenib. In a previous study, the ridge regression model was trained on the GDSC cell line data and evaluated by 10-fold cross-validation [36]. We observed a significant difference in the estimated IC_50_ values between subtype 1 and subtype 2 for paclitaxel and sorafenib, whereas subtype 1 showed high sensitivity to commonly administered chemotherapies (*p* = 0.0013 for paclitaxel and *p* = 0.031 for sorafenib) (Figure 5C).

### 2.4. Classifier Construction and Validation

**Figure 6:**
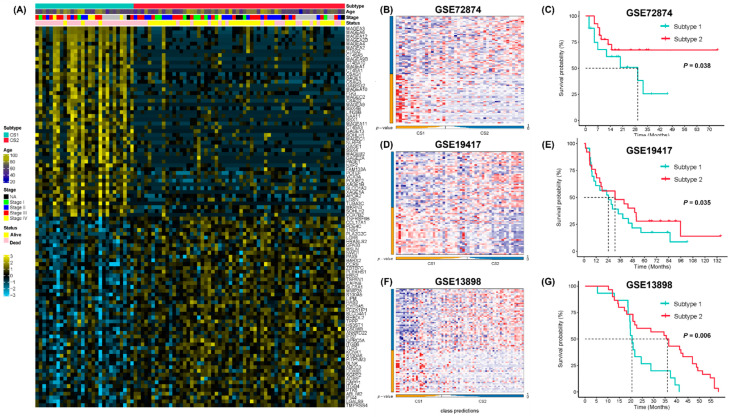
Validation of EAC subtyping using external datasets GSE72874, GSE19417, and GSE13898. (**A**) Heatmap of subtype-specific signature genes for identified subtypes in TCGA-EAC. (**B**) Heatmap of subtype-specific signature genes in GSE72874. (**C**) Kaplan–Meier survival curve of the EAC subtypes in GSE72874. (**D**) Heatmap of subtype-specific signature genes in GSE19417. (**E**) Kaplan–Meier survival curve of the EAC subtypes in GSE19417. (**F**) Heatmap of subtype-specific signature genes in GSE13898. (**G**) Kaplan–Meier survival curve of the EAC subtypes in GSE13898.

The external validation datasets GSE72874, GSE19417 and GSE13898 included 44, 48 and 45 EAC samples with overall survival information, respectively. Gene expression data and clinical information were then obtained from the GEO database to validate the prognostic value of the two EAC subtypes. The top 50 subtype-specific upregulated signature genes with the largest log2 fold-change values were selected. The NTP algorithm was then used to classify the validation datasets (Figure 6A). In GSE72874, 17 and 27 samples were identified as subtype 1 and subtype 2, respectively (Figure 6B). In GSE19417, 23 and 25 samples were identified as subtype 1 and subtype 2, respectively (Figure 6D). In GSE13898, 15 and 30 samples were identified as subtype 1 and subtype 2, respectively (Figure 6F). We compared the overall survival between the two subtypes in the GSE72874, GSE19417, and GSE13898 datasets and found that subtype 2 showed better survival consistently (log-rank test, *p* = 0.038, Figure 6C; *p* = 0.035, Figure 6E; *p* = 0.006, Figure 6G).

## 3. Discussion

Studies on the molecular subtyping of EAC have mainly been based on overall expression profiles and somatic mutation information [7,37,38]. The importance of TME in cancer is gaining greater recognition, especially in the context of cancer immunotherapy [39]. It remains necessary to characterize the heterogeneous TME of EAC and reveal its association with therapeutic responses in order to improve clinical treatments. This study, for the first time, identified response-related molecular and TME subtypes of EAC by integrating available public genomic and pharmaceutical data, thus providing new guidance for precise treatment of EAC.

The upregulated differentially expressed protein-coding genes in subtype 2 include atypical chemokine receptor (ACKR1) [40], which functions as a tumor suppressor in several cancers and plays an important role in transporting chemokines across cells to aid leukocyte transmigration [41]. Another upregulated gene, EPCAM, has also been identified as a marker for cancer stem cells [42]. DEGs upregulated in subtype 1 included chemokine CCL3 [43], which is frequently overexpressed in tumor sites, leading to microenvironmental dysfunction. In addition, overexpression of TNFAIP6 was also found to increase with tumor invasion and lymph node metastasis in gastric cancer [44]. Apolipoprotein C2 (APOC2) is a lipoprotein lipase activator involved in fatty acid metabolism [45]. Most differentially expressed lncRNAs, such as AL121906.2 [46], AC092910.3 [47], AC005332.4 [48], and CASC9 [49], were upregulated in EAC subtype 1 and had been previously reported as prognostic biomarkers in multiple tumors. These findings highlighted the intrinsic molecular differences between the two EAC subtypes. Further GO annotation indicated that the two subtypes showed distinct patterns of immunologic profiles.

Although the results of ICI therapy for esophageal cancer are promising, the proportion of patients who are responsive to ICIs is still low. In this study, we characterized EAC patients based on differentially expressed genes and TME signatures relevant to immunotherapy response. Using the unsupervised integrative NMF method, two EAC subtypes with distinct prognoses were identified. EAC subtype 2 showed enriched features of immune cells (CD8^+^/CD4^+^ T cells, B cells) and up-regulated immune-related signaling pathways. EAC subtype 2 also had higher levels of macrophages, fibroblast infiltration, and higher enrichment scores for antigen-presenting and metabolism-relevant signatures, suggesting that subtype 2 might be more sensitive to immunotherapy or immune checkpoint inhibition. Interestingly, the most upregulated genes in EAC subtype 1 were from the melanoma antigen gene (MAGE) family (Figure 6A), which have potential value as targets of immunotherapies, since these proteins are also cancer/testis antigens [50]. MAGEs have been reported to promote tumor growth via a variety of pathways, resulting in more aggressive, metastatic tumors with higher risk of recurrence [51]. Further studies on MAGE function may facilitate the development of immunotherapy in EAC [52].

Using a drug-sensitivity database and a drug-response model, we predicted that EAC subtype 1 could be more sensitive to commonly used chemotherapies. The results further implicate that subtype 1 may benefit from a combination of chemotherapy and immunotherapy. Although we did not find any significant differences in the response rate between the two subtypes according to the TIDE scores, the SubMap method suggested that EAC subtype 2 was more likely to respond to anti-PD-1 therapy, in line with the TME characteristics of this subtype.

Briefly, we sought here to provide a comprehensive understanding of the molecular and TME characteristics of EAC to facilitate precise classification and treatment selection. The molecular and TME differences between the EAC subtypes may guide clinical treatment selection. There are, however, some minor limitations to this study. It remains challenging to predict immunotherapy response unless EAC immunotherapy trial data are available. The small sample size also led to difficulties in precisely detecting differences in clinicopathological traits. Moreover, the subtype classifier proposed here requires further verification with clinical samples at the protein level.

## 4. Materials and Methods

### 4.1. Data Source and Processing

The publicly available esophageal adenocarcinoma cancer datasets (GSE72874, GSE92396, GSE13898, and GSE19417) were downloaded from the Gene Expression Omnibus database (GEO). These patients did not receive any treatment before surgery. All GEO datasets were log2-transformed, annotated, and quantile-normalized. The probe-level data were converted into gene symbols according to the corresponding annotation file. The maximum value was selected when multiple probes corresponded to the same gene symbol. The potential batch effects between different experiments were removed using the R package sva. Specifically, 54 peritumor esophageal and 135 esophageal adenocarcinoma samples from GSE72874, GSE92396, and GSE13898 were combined to identify differentially expressed genes in EAC.

The RNA-sequencing (RNA-seq) profiles and patient survival information from the pan-cancer data in the TCGA database were downloaded from UCSC-XENA (https://xenabrowser.net/, Accessed on 20 October 2021). Finally, 88 samples diagnosed as “Esophagus Adenocarcinoma” in the TCGA-ESCA cohort were included as a training cohort to filter prognostic related protein-coding and long non-coding RNAs for molecular subtyping. GSE13898, GSE72874, and GSE19417 were retrieved to check the clinical relevance of the identified molecular subtypes. A flow chart to illustrate the study design was shown in Figure 1.

### 4.2. Selection of Differentially Expressed Genes with Prognostic Value

Based on the discovery GEO cohort of EAC patients, differentially expressed genes (DEGs), including protein-coding and long non-coding RNAs, in 135 esophageal adenocarcinoma and 54 normal esophageal samples were identified using the R package limma (*p*-value < 0.05, |log2FC| > 2). Since the discovery cohort lacked survival information, 276 DEGs were further screened after overlapping with DEGs identified in the TCGA-EAC tumor samples (*n* = 88) using univariable Cox proportional hazards regression (*p* < 0.05). We selected overall survival (OS) as the survival endpoint.

### 4.3. Estimation of Immune Infiltration

To avoid potential bias and pitfalls of different TME cell deconvolution algorithms [53], we applied 8 deconvolution methods incorporated in the R package IOBR [54] to characterize the TME composition of EAC, including CIBERSORT [55], xCell [56], TIMER [57], MCPCounter [58], EPIC [59], ESTIMATE [60], quanTIseq [61], and immunopheno score (IPS) [62]. The deconvolution results obtained using the above methods were calculated for the TCGA-EAC training cohort as well as the validation datasets GSE72784 and GSE19417. TME scores were used in the following clustering procedure together with gene expression scores to identify EAC subtypes.

### 4.4. Identification and Validation of EAC Subtypes

Before performing NMF, candidate DEGs and TME scores with absolute median difference (MAD) values less than 0.5 were excluded. We then used selected features to classify EAC samples using an integrative non-negative matrix factorization (NMF) method (IntNMF) [63]. We chose IntNMF since it outperformed other algorithms in a benchmark study on multi-omics data integration [64]. Specifically, the NMF algorithm factorized the gene expression matrix *A* of *N* genes and *M* samples into 2 non-negative matrices, *W* and *H* (i.e., A≈W×H), where the sizes of *W* and *H* are *N* × *k* and *k* × *M*, respectively. The outputs of matrix *A* were then integrated into k clusters through iterative decomposition. IntNMF considered shared factors across all ‘omics’ datasets (i.e., gene expression and TME scores in this study). IntNMF extended NMF by integrating the multiple data matrix *A^i^* and estimating the common basis matrix *W* and coefficient matrices *H^i^* such that $A_{n\times m_{i}}^{i}\approx W_{n\times k}H_{k\times m_{i}}^{i}$, where *W* and *H^i^* are all non-negative The clustering prediction index and gap statistics were used to determine the optimal number of clusters (k). We also applied the other 9 clustering algorithms and calculated a consensus matrix to verify clustering robustness. Silhouette scores were calculated to quantify and visualize similarity among clusters using the R package MOVICS [65]. The top 50 specifically expressed signature genes in each EAC subtype were identified based on differential expression analyses against the other subtype using the R package DESeq2 (Benjamini–Hochberg-adjusted *p*-value < 0.05, |log2FC| > 2). Nearest template prediction (NTP) classifiers were trained using the TCGA EAC cohort for class predictions for each sample using the top 50 signature genes [66].

### 4.5. Molecular and TME Characterization between EAC Subtypes

Gene set enrichment analyses (GSEAs) were performed with Gene Ontology (GO) biological process annotation using the R package clusterProfiler. The enrichment scores of molecular pathways were evaluated using single-sample gene set enrichment analysis using the R package GSVA [67].

### 4.6. Therapeutic Response Prediction

The Tumor Immune Dysfunction and Exclusion (TIDE) algorithm and subclass mapping (SubMap) were used to predict responses to immune checkpoint blockade [68,69]. TIDE scores were calculated for each sample, a score > 0 indicating a lower likelihood of responding to ICIs. Meanwhile, the SubMap algorithm of GenePattern (https://cloud.genepattern.org/, Accessed on 21 December 2021) was applied to predict response to immunotherapy in the identified EAC subtypes (nominal *p* < 0.05 or Bonferroni correction < 0.05). The immunotherapy response data were log2-transformed NanoString-normalized gene expression data for 47 melanoma patients treated with CTLA-4 and PD-1 checkpoint blockade [35].

We used a ridge regression model in the R package pRRophetic [36] to predict chemotherapeutic responses for the TCGA-EAC cohort and examined differences in drug sensitivity (half-maximal inhibitory concentration (IC_50_)) among different subtypes. The lower the IC_50_ value of the drug, the greater the drug’s potency in inhibiting cell growth. This predictive model was trained on mRNA gene expression and drug response data of CCLs in the Genomics of Drug Sensitivity in Cancer (GDSC) and was previously evaluated by 10-fold cross-validation.

### 4.7. Statistical Analysis

Statistical analyses and visualizations were performed using R (version 4.0.1, R Core Team, Vienna, Austria). We used the R package survminer and survival to perform a univariate Cox regression analysis. A survival analysis was carried out using Kaplan–Meier methods with log-rank tests to determine statistical differences. For all statistical analyses, Fisher’s exact test was used for categorical data, the Mann–Whitney test was used for continuous data, and a two-tailed *p*-value less than 0.05 was considered statistically significant.

## 5. Conclusions

Overall, we identified two molecular subtypes of EAC and constructed a classification system that might aid in predicting the prognosis and therapy response of patients, especially those more likely to respond to immunotherapy. We also suggest MAGE family genes as potential targets for those patients who are unlikely to benefit from current immune checkpoint blockade therapy.

## Data Availability

All datasets utilized in this study are publicly available and were accessed from several repositories. The bulk microarray gene expression and subject data are available in the Gene Expression Omnibus (GEO). The bulk RNA-seq gene expression and subject data from the TCGA ESCA project are available through the Genomic Data Commons: https://gdc.cancer.gov/access-data/, accessed on 30 October 2021.

## Supplementary Materials

The following supporting information can be downloaded at: https://www.mdpi.com/article/10.3390/ph15050605/s1, Figure S1: Subgroup analyses revealed by Kaplan-Meier survival curve; Figure S2: Kaplan-Meier survival curve of TME scores in the TCGA-EAC cohort.

## Abbreviations

EAC	Esophageal adenocarcinoma
ESCC	Esophageal squamous cell carcinoma
CCL	Cancer cell lines
TCGA	The Cancer Genome Atlas
GEO	Gene Expression Omnibus dataset
DEG	Differentially expressed gene
GDSC	Genomics of Drug Sensitivity in Cancer
TIDE	Tumor Immune Dysfunction and Exclusion
