# Supplementary material for: Identification of Immune Subtypes of Esophageal Adenocarcinoma to Predict Prognosis and Immunotherapy Response
Source: Pharmaceuticals (Basel). 2022 May 14;15(5):605. doi: 10.3390/ph15050605 (PMC9144862; doi:10.3390/ph15050605)
Supplement: Supplementary file 1 [file pharmaceuticals-15-00605-s001.zip › pharmaceuticals-1674294-supplementary.pdf]

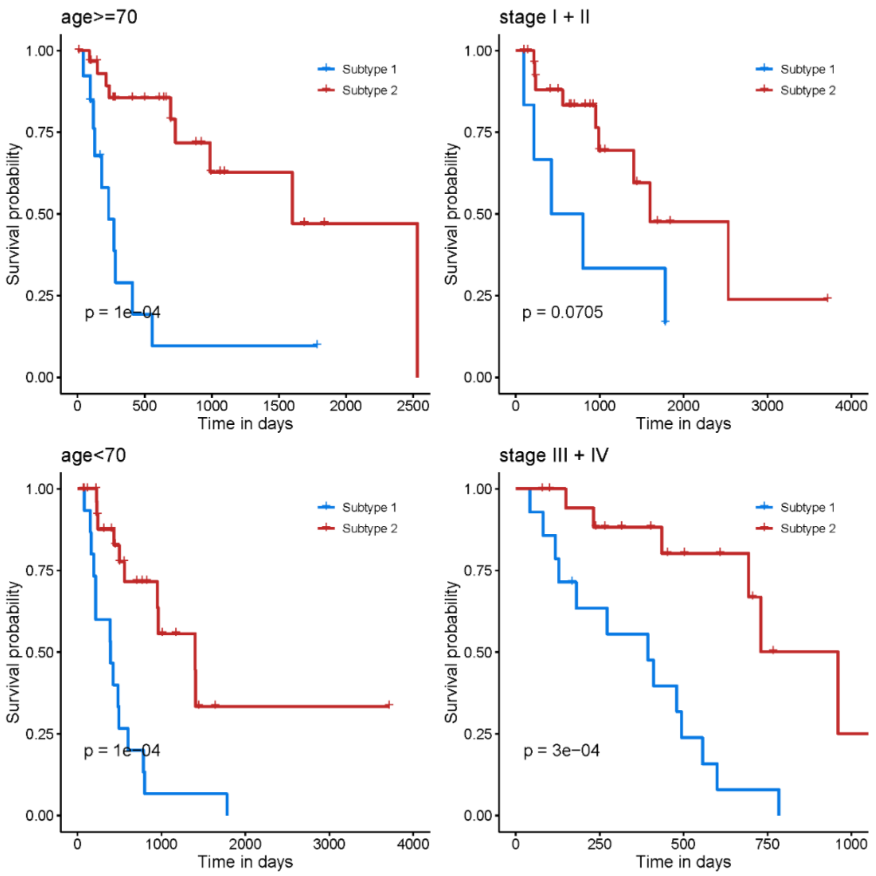

Supplementary Fig. S1 Subgroup analyses revealed by Kaplan-Meier survival curve.

Stratified survival analyses based on the clinicopathological features age and histological stage for the EAC subtypes in the TCGA-EAC cohort.

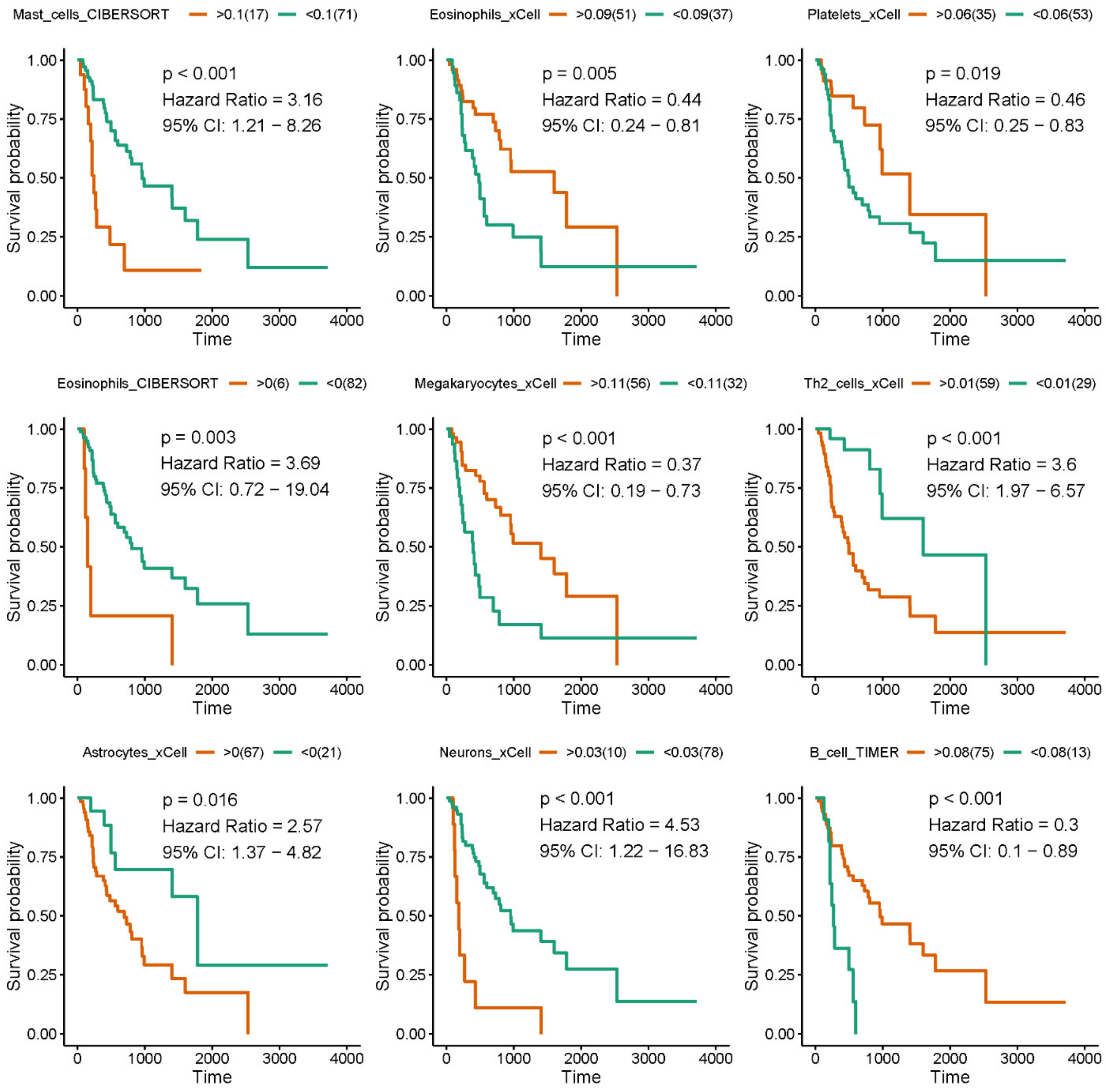

Supplementary Fig. S2 Kaplan-Meier survival curve of TME scores in the TCGA-EAC cohort.
